# In Vivo Silencing of Genes Coding for dTip60 Chromatin Remodeling Complex Subunits Affects Polytene Chromosome Organization and Proper Development in *Drosophila melanogaster*

**DOI:** 10.3390/ijms22094525

**Published:** 2021-04-26

**Authors:** Yuri Prozzillo, Stefano Cuticone, Diego Ferreri, Gaia Fattorini, Giovanni Messina, Patrizio Dimitri

**Affiliations:** 1 Dipartimento di Biologia e Biotecnologie “Charles Darwin” Sapienza Università di Roma, 00185 Rome, Italy; yuri.prozzillo@uniroma1.it (Y.P.); cuticone.1249495@studenti.uniroma1.it (S.C.); ferreri.1656912@studenti.uniroma1.it (D.F.); fattorini.1701139@studenti.uniroma1.it (G.F.); 2 Istituto Pasteur Italia, Fondazione Cenci-Bolognetti, 00185 Rome, Italy

**Keywords:** *Drosophila melanogaster*, polytene chromosomes, chromatin remodeling, position effect variegation, epigenetic silencing

## Abstract

Chromatin organization is developmentally regulated by epigenetic changes mediated by histone-modifying enzymes and chromatin remodeling complexes. In *Drosophila melanogaster*, the Tip60 chromatin remodeling complex (dTip60) play roles in chromatin regulation, which are shared by evolutionarily-related complexes identified in animal and plants. Recently, it was found that most subunits previously assigned to the dTip60 complex are shared by two related complexes, DOM-A.C and DOM-B.C, defined by DOM-A and DOM-B isoforms, respectively. In this work, we combined classical genetics, cell biology, and reverse genetics approaches to further investigate the biological roles played during *Drosophila melanogaster* development by a number of subunits originally assigned to the dTip60 complex.

## 1. Introduction

ATP-dependent chromatin remodeling complexes are multiprotein cellular machinery that use ATP energy to modify chromatin organization by sliding or displacing nucleosomes and exchanging histones. Such changes alter histone–DNA interactions making nucleosomal DNA more accessible to specific factors involved in transcription, replication and repair [1,2].

ATP-dependent chromatin remodeling complexes generally consist of evolutionary conserved subunits, one of which carries an ATPase domain. They are subdivided into four different families called SWI/SNF, ISWI, NURD/Mi-2/CHD, and INO80 [1,2].

Among the INO80 family, the *Drosophila melanogaster* Tip60 complex (dTip60) was found to be made up by 16 core subunits (ACT87E, BAP55, BRD8, DOMINO, DMAP1, EAF6, E(PC), GAS41, ING3, MRG15, MRGBP, NIPPED-A, PONTIN, REPTIN, and YL-1) [3]. Further studies have identified YETI as an additional subunit of dTip60 complex [4,5,6]. In accord, YETI is orthologous to SWC5, a subunit of the yeast SWR1 chromatin remodeling complex [7].

The dTip60 complex was suggested to be required for the replacement of acetylated phospho-H2A by unmodified H2A.V via DOMINO (DOM) ATPase [3,8]. In addition, dTip60 complex has been proposed to control the deposition of H2A.V on chromatin [4,5,6,8,9], similarly to the function played by evolutionary related complexes in yeast and human cells. Indeed, dTip60 complex subunits shares high sequence conservation with those of yeast SWR1 and human SRCAP and P400/Tip60 complexes, which govern H2A-H2AZ exchange into chromatin [2,10,11,12].

Recently, mass-spectrometry analyses carried out in *D. melanogaster* cell lines provided evidence that two DOMINO isoforms, DOM-A and DOM-B, define two different chromatin remodeling complexes, called DOM-A.C and DOM-B.C, characterized by a different subunit composition [13,14]. Among the subunits that have been assigned to dTip60 complex [3], nine are shared by both DOM-A.C and DOM-B.C, four, (E(PC), ING3, NIPPED-A, and TIP60) are specific for DOM-A.C, while ACT87E, EAF6 e YETI [2,10,11,12] were not found in DOM-A.C and DOM-B.C. Finally, ARP6, not included in the dTip60 complex, was identified as a subunit of DOM-B.C [13,14]. Moreover, DOM-A.C was suggested to be the functional equivalent of the yeast NuA4.C, which acetylates the H4 N-terminus [15], while DOM-B.C is responsible for H2A.V deposition in an ATP-dependent manner.

*Drosophila melanogaster* H2A.V is a structural and functional chimera of H2A.Z and H2A.X [9]. Like the H2A.X, H2A.V is phosphorylated upon DNA double strand breaks to mark the DNA lesions, thus stimulating DNA repair machinery and promoting the formation of accessible DNA conformation [2].

Previous studies showed that DOMINO, NIPPED-A, PONTIN, TIP60 and YETI subunits are essential for development and chromatin regulation [4,16,17,18,19], while BAP55 functions through the dTIP60 complex to regulate olfactory projection neuron dendrite targeting [20]. For other subunits, such as EAF6 and DMAP1, few studies are available. EAF6 was found to play a role in H3K23 acetylation and DMAP1 is a DNA methyltransferase 1-associated protein involved in the modulation of the innate immune response pathway in *D. melanogaster* [21,22,23]; however, it is not clear whether they are essential for *D. melanogaster* viability and development.

Here, we combined classical genetics, cell biology and reverse genetics approaches to further investigate the biological functions played during *D. melanogaster* development by a number of subunits originally assigned to the dTip60 chromatin remodeling complex.

## 2. Results

### 2.1. Distribution of DMAP1, DOM-A, DOM-B, MRG15, TIP60, and PONTIN on Polytene Chromosomes

First, we analyzed the distribution of DOM-A, DOM-B, TIP60, MRG15, DMAP1, and PONTIN on polytene chromosome by immunofluorescence microscopy (IFM).

**Figure 1:**
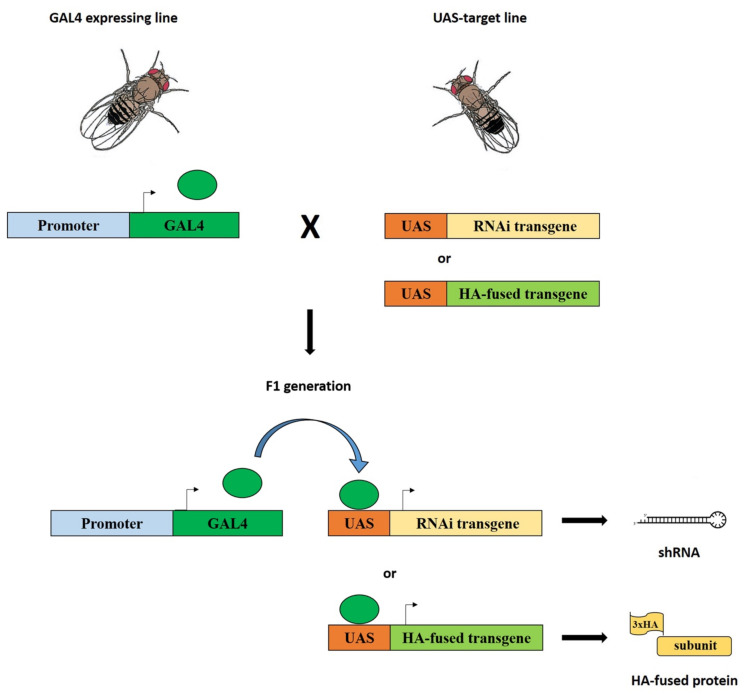
Silencing of genes coding for dTip60 complex subunits and expression of HA-fused subunits using the GAL4-UAS system. Flies homozygous for a given *GAL4* transgene (driver) under control of specific regulatory sequences were crossed to flies carrying a specific *UAS transgene* (target): *UAS-RNAi* or *UAS-HA*. In the F1 progeny from these crosses, both elements, driver and target, were present together in the same individuals and the expression *of UAS-RNAi* or *UAS-HA* transgenes was activated by GAL4, which, in turn, give rise to the formation of shRNAs (short harpin RNAs) or HA-fused subunits, respectively.

**Figure 2:**
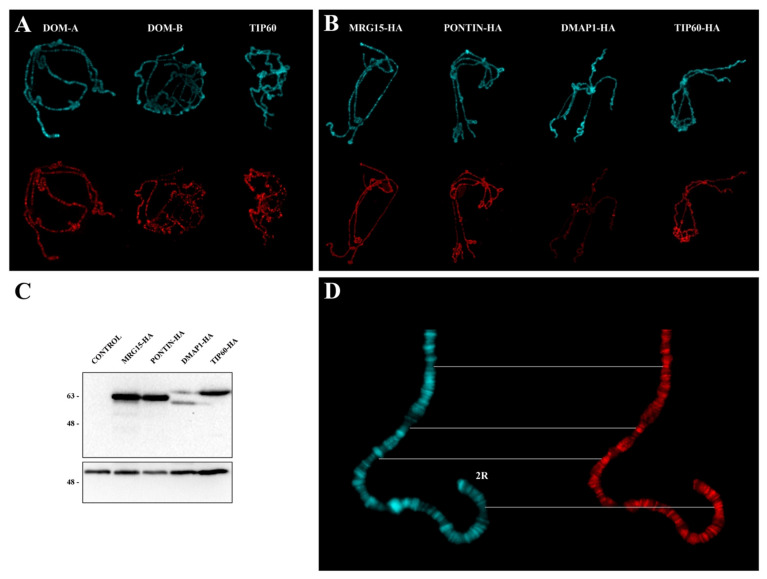
Polytene chromosome distribution of endogenous and HA-fused subunits. (**A**) Distribution of endogenous DOM-A, DOM-B and TIP60. DAPI staining (blue), immunostaining (red). (**B**) Distribution of MRG15, PONTIN, DMAP1, and TIP60 HA-fusion proteins. (**C**) Western blotting on protein extracts from third instar larvae showing the expression of HA-fusion proteins following activation. No expression was detected in not activated controls. Numbers indicate the molecular weight in kD. (**D**) Example of PONTIN-HA distribution along the distal portion of chromosome 2R: The signals tend to be located at the interbands. DAPI staining (blue), immunostaining (red).

The distribution of endogenous DOM-A, DOM-B, and TIP60 was studied by indirect immunofluorescence with specific antibodies (Material and Methods). To detect MRG15, DMAP1 and PONTIN subunits, in the absence of specific antibodies, we made use of strains carrying the following UAS-HA-tagged transgenes: *DMAP1-HA, MRG15-HA*, and *PONTIN-HA*. Using the GAL4-UAS system [24], flies homozygous for a given *UAS-HA*-tagged transgene were crossed to flies carrying a *Tub-GAL4* driver to express the corresponding HA-fusion protein in the F1 progeny (Figure 1; Materials and Methods;). The distribution of each HA-fusion proteins was assessed on salivary gland polytene chromosomes. The distribution of TIP60 was also studied using a line carrying a specific *UAS-TIP60-HA*-tagged transgene. The results of these experiments showed that both the endogenous subunits and the expressed HA-fused subunits are distributed at multiple sites on polytene chromosomes (Figure 2A–C). Interestingly, all the proteins tested tend to be located in the polytene chromosome interbands (see the example in Figure 2D), as already reported for TIP60 and BAP55 [18,25].

### 2.2. RNAi Silencing of Genes Coding for dTip60 Complex Subunits Affects Individual Viability

Previous studies showed that dTip60 complex subunits are essential for fly viability. Ubiquitous knockdown of TIP60 protein is lethal [18], while loss-of-function *Domino* and *Yeti* mutants showed prolonged larval development followed by lethality before pupation together with the presence of large melanotic masses in the larval hemocoel [4,16]. The loss-of-function of PONTIN induced JNK activation and initiated JNK-mediated cell death [19].

Here, we performed *in vivo* RNAi-mediated silencing of genes coding for the subunits of interest using the GAL4-UAS system [25]. The following subunits were taken into consideration: BAP55, DMAP1, DOM-A/DOM-B, EAF6, E(PC), GAS41, MRG15, NIPPED-A, PONTIN, REPTIN, TIP60, YETI, and YL-1. For each subunit, flies homozygous for a given *UAS-RNAi* transgene were crossed to flies carrying a *Tub-GAL4* driver to express the corresponding shRNA (short harpin RNA) in the F1 progeny. This, in turn, produced the ubiquitous silencing of the gene coding for the subunits of interest from earliest stages of development (Figure 1; Materials and Methods;). In the case of *UAS-Domino RNAi* transgene (VDRC line 7787), the expressed shRNA induced the simultaneous silencing of both *Domino* transcripts coding for DOM-A and DOM-B isoforms.

As shown in Table 1, in vivo silencing of the genes tested affected the viability with a lethal phase ranging from early larva to late pupal stage. These results confirmed and extended previous findings showing that the dTip60 subunits are essential for fly viability.

### 2.3. RNAi Silencing of Genes Coding for dTip60 Complex Subunits Affects Higher-Order Organization of Salivary Gland Polytene Chromosomes

**Figure 3:**
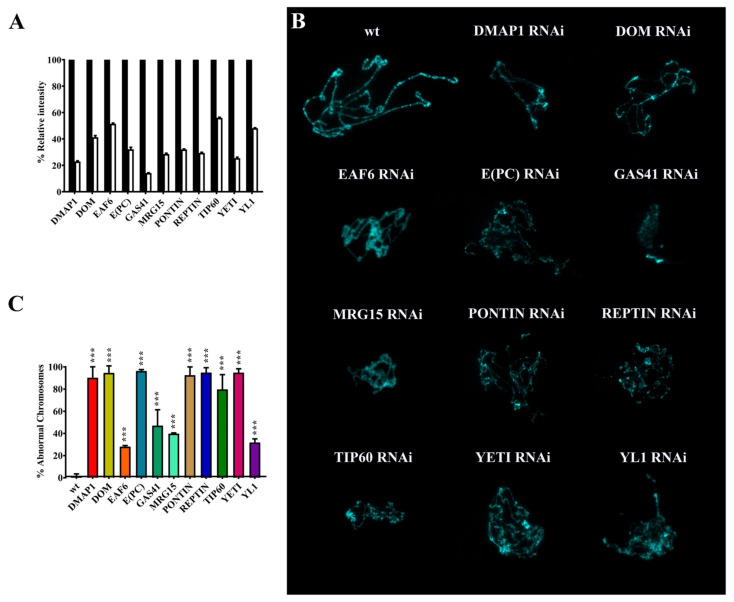
Polytene chromosome defects after silencing of genes coding for dTip60 complex subunits. (**A**) Semi-quantitative PCR of RNA extracted from salivary glands showed significant silencing of the genes encoding the subunits of interest. (**B**) Examples of salivary gland polytene chromosome defects. (**C**) Quantification of polytene chromosome defects. *p* value <0.0005 (***), compared with the control group.

We analyzed the silencing effect of genes coding twelve subunits (DMAP1, DOM-A/DOM-B, EAF6, E(PC), GAS41 MRG15, PONTIN, REPTIN, TIP60, YETI, and YL-1) on higher-level chromatin organization of salivary gland polytene chromosomes. Since silencing of most genes caused early lethality (Table 1), we circumvented this problem using a *Tub-GAL4-GAL80^ts^* driver. For each subunit, flies homozygous for a given *UAS-RNAi* transgene were crossed to flies carrying an inducible *Tub-GAL4-GAL80^ts^* driver. The temperature-sensitive *GAL80^ts^* allowed us to induce the silencing of the genes of interest in a specific stage of larval development. The F1 progeny of each cross was kept at 18 °C (permissive temperature) until the second larval instar stage and subsequently transferred to 29 °C to activate the expression of a given *UAS-RNAi* transgene. Salivary glands were extracted from the recovered third instar larvae and used to perform polytene chromosome squashes. The RNAi efficiency was determined by semi-quantitative PCR of RNA extracted by salivary glands and significant silencing of the genes coding for the subunits of interest was observed (Figure 3A, Table 2). Again, the expression of *UAS-Domino*-*RNAi* transgene induced the simultaneous silencing of both *Domino* transcripts coding for DOM-A and DOM-B isoforms.

As shown in Figure 3B,C and Table 3, the silencing of the genes coding for the subunits under investigation led to significant alterations of higher-order organization of polytene chromosomes. Chromosomes appeared smaller and with a thin and disorganized structure.

### 2.4. RNAi Silencing of Genes Coding for dTip60 Complex Subunits Affects Eye Morphology and Differentiation

**Figure 4:**
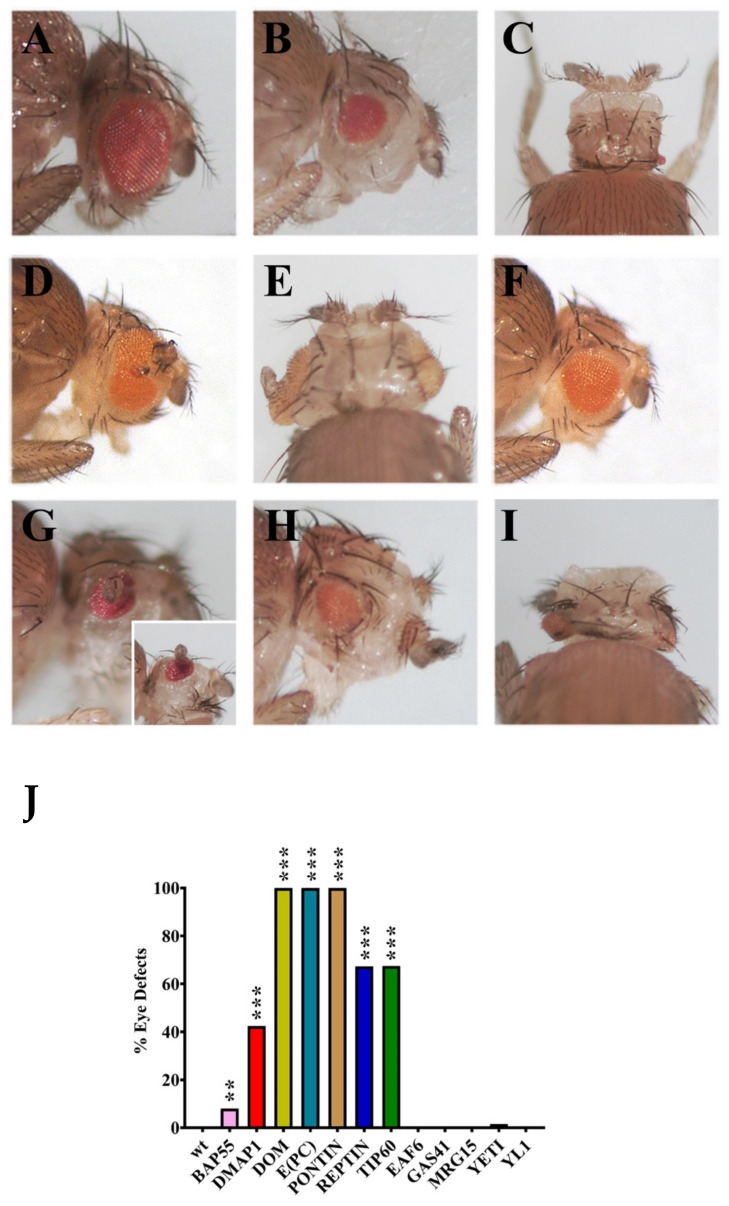
Eye morphology and differentiation defects after silencing of genes coding for dTip60 complex subunits. (**A**) Wild-type control; (**B**,**C**) silencing of DMAP1 coding gene caused eye reduction and microcephaly, respectively; (**D**,**E**) simultaneous silencing of DOM-A and DOM-B caused eye reduction and formation of extra-growths; (**F**) eye reduction after E(PC) silencing; (**G**) PONTIN silencing caused eye reduction and formation of extra-growth; (**H**) strong eye reduction; and (**I**) microcephaly after REPTIN silencing; (**J**) quantification of eye defects. *p* value <0.005 (**) or *p* value <0.0005 (***), compared with the control group.

Next, we studied the phenotypic effects caused by RNAi silencing of genes coding for dTip60 complex subunits on eye morphology and differentiation. In these experiments the following subunits were considered: BAP55, DMAP1, DOM-A/DOM-B, E(PC), EAF6, GAS41, MRG15, PONTIN, REPTIN, TIP60, YETI, and YL-1. For each subunit, flies homozygous for a given *UAS-RNAi* transgene were crossed to flies homozygous for an ey-GAL4 transgene (Figure 1; Materials and Methods;), which is abundantly expressed in the fly eye thanks to the *eyeless* gene regulatory sequences; the F1 progeny was scored for defects of eye morphology/differentiation. The results of this analysis are shown in Figure 4 and Table 4.

We observed two categories of eye alterations: (1) Eye size reduction and (2) eye malformations with the appearance of extra-growths, often accompanied by head size reduction. The effects differ between genes. In particular, silencing of DMAP1, DOM-A/B, PONTIN and REPTIN genes coding caused the most drastic phenotypes with high penetrance in the offspring (Table 4), including eye size reduction and formation of ectopic structures within the region that normally differentiates into eye. Notably, silencing of DMAP1 and REPTIN coding genes also produced the appearance of microcephalic phenotypes (Figure 4C,I). Silencing of E(PC) and TIP60 coding genes caused a strong effect on eye-size reduction, while minor defects with lower penetrance were observed for BAP55 and YETI. Finally, no effects on eye morphology and differentiation were seen after silencing of EAF6, GAS41, MRG15 and YL-1 coding genes. This suggests that some dTip60 subunits play crucial roles in eye differentiation, while other subunits may be not relevant, or even not essential, for eye development.

### 2.5. Mutations of Bap55, Eaf6, and Pontin Genes Are Dominant Suppressor of PEV

Position effect variegation (PEV) is a well-known example of epigenetic silencing resulting in the stochastic inactivation of euchromatic genes juxtaposed to heterochromatin by chromosome aberrations or transposition [26]. Testing the effect of mutations in the genes coding the TIP60 subunits on PEV can contribute to assess their role in the epigenetic regulation of gene expression. Previous findings showed that mutations in *Domino, Reptin, E(Pc), Mrg15*, and *Gas41* genes are dominant suppressor of PEV [27,28,29,30]. Here, we tested the effect of mutations in *Bap55, Eaf6* and *Pontin* genes on the variegated eye phenotype of *In(1)wm4*. *In(1)wm4* is a classical variegated rearrangement where the *white+* gene is moved at close contact to the constitutive heterochromatin of the X chromosome by a paracentric inversion.

*In(1)wm4*/*In(1)wm4* homozygous females were mated in separated crosses to *w^1118^;Bap55^EY15967^/T(2;3)CyTb, w^1118^, Eaf6^d06605^/T(2;3)CyTb* or *w^1118^; Pontin^5.1/^T(2;3)CyTb* males and the amount of red eye pigment was assessed in *Cy+ Tb+* F1 male progeny (Material and Methods). As positive controls, *Su(var)205/CyTb* and *Dom3/CyTb* lines were used. The *Su(var)205* wild-type gene encodes the HP1 protein, a multi-functional epigenetic regulator involved in heterochromatin formation and *Su(var)205* mutations are strong dominant suppressors of PEV [26,31]. As previously recalled, *Domino* mutant alleles are also dominant suppressors of PEV [28].

**Figure 5:**
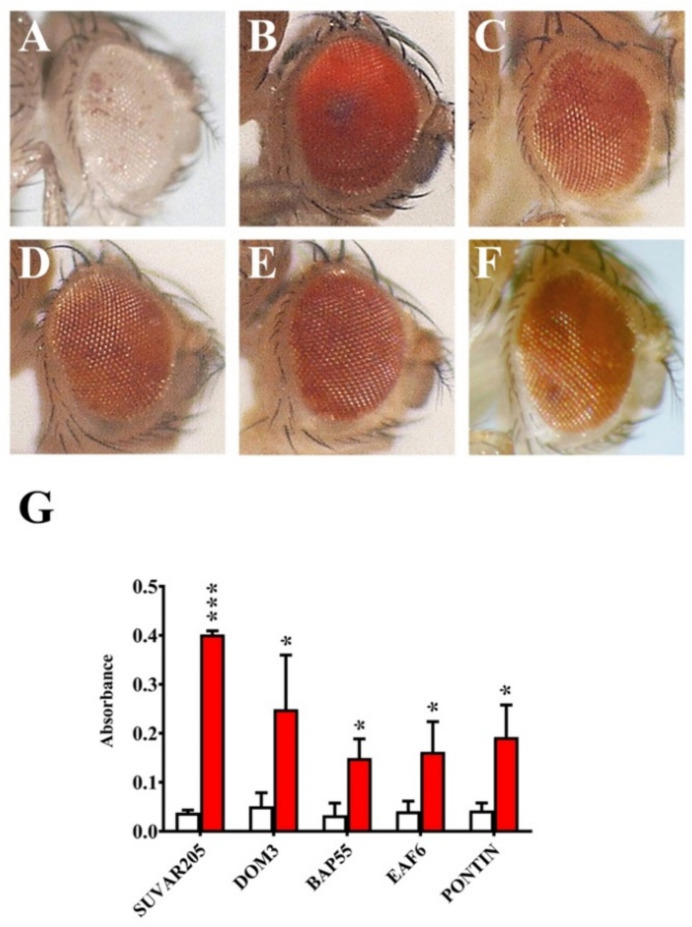
Dominant suppression of *In(1)wm4* variegation by mutations in *Bap55, Eaf6*, and *Pontin* genes. (**A**) Standard *In(1)wm4* variegated phenotype (control); (**B**) Dominant suppression of *In(1)wm4* variegation by *Su(var2)05* and (**C**) *Dom3* mutants (positive controls). Dominant suppression of *In(1)wm4* variegation by (**D**) *Bap55^EY1596^*, (**E**) *Eaf6^d06605^*, and (**F**) *Pontin^5.1^* mutant alleles. (**G**) Quantification of PEV suppression in terms of O.D. Red histograms = eye pigment in presence of the tested mutations; white histograms = controls. *p* value <0.05 (*) or *p* value <0.0005 (***), compared with the control group.

Among the tested mutations, *Pontin^5.1^* is a deletion [32], while *Bap55^EY1596^* and *Eaf6^d06605^* are due to a *Pw+* transposon insertion (see FlyBase) which confers a light-yellow eye color, a background which does not interfere with the quantification of the red eye pigment in the experimental samples (Figure S1). The results of these experiments are shown in Figure 5 and Table 5. As expected, the *In(1)wm4* variegation was suppressed by *Su(var)205* and *Dom3* mutations. Most importantly, we found that *Bap55^EY1596^*, *Eaf^d06605^*, and *Pontin^5.1^* alleles were dominant suppressor of *In(1)wm4* variegated phenotype (Figure 5A,B; Table 5). Interestingly, the *Dom3* allele failed to suppress the variegated eye phenotype of *7m27*, a variegated *Pw+* insertion into the Y chromosome telomeric region [33], which by contrast was efficiently suppressed by *Su(var)205)* allele (Figure S2).

## 3. Discussion

In this work we investigated the effects caused by in vivo silencing of genes coding for dTip60 complex subunits on individual viability, chromosome organization, eye development and epigenetic silencing.

It was previously found that BAP55 and TIP60 subunits tend to be localized to the polytene chromosome interbands [18,24]. We confirmed such localization for TIP60 and found a similar behavior for DOM-A, DOM-B, PONTIN-HA, MRG15-HA, and DMAP1-HA (Figure 2). The polytene chromosome interbands are DAPI-negative regions that are usually associated with open chromatin regions characterized by the presence of RNA polymerase II, chromatin remodeling complexes and proteins that recognize the origins of DNA replication [34,35]. It is possible that occupancy of these regions by the tested subunits could facilitate the action in switching off or on of group of genes during *D. melanogaster* development or making the intervention in DNA damage response timely and efficiently. It has indeed been found that a reduction of the levels of the TIP60 protein in *D. melanogaster* caused up or down regulation of group of genes, implying a role in both gene activation and repression [18].

It has been shown that the lack of dTip60 complex subunits affect individual viability [4,16,18,19]. We found that silencing of genes coding for the subunits under investigation caused developmental arrest, with the lethal phase ranging from early larval stage to late pupal stage (Table 1). These results indicated the tested subunits are essential for viability and proper development of *D. melanogaster*. It is possible that the lack of a single subunit could compromise the formation/function of the whole complex. It is also possible that some subunits may perform individual functions independently from the complex as a whole.

It has been suggested that MRG15 plays a role in chromatin compaction in *D. melanogaster* by recruiting the activity of condensins [36]. In addition, the loss of YETI strongly affects polytene chromosome organization in *D. melanogaster* [4] and a similar defect was observed on mitotic chromosomes by the depletion of CFDP1, the human ortholog of YETI [37], a subunit of the SRCAP complex. Here, we found that silencing of genes coding for the tested subunits strongly affected polytene chromosomes that appeared smaller and with a thin and disorganized structure where the normal pattern of banding is not distinguishable (Figure 3, Table 3). These defects are consistent with chromatin condensation defects associated with a failure in DNA replication during polytenization. Intriguingly, chromatin remodeling factors were found to colocalize with origin recognition complex (ORC) 2 subunit, at the level of polytene chromosome interbands [34].

Silencing of the genes coding for twelve dTip60 subunits also affected eye development. In particular, silencing of DMAP1, DOM-A and DOM-B, PONTIN, E(PC), and REPTIN coding genes caused drastic defects of eye morphogenesis and differentiation, including the formation of extra-growths and microcephalic phenotypes (Figure 4, Table 4). These results suggest that the tested subunits play crucial roles in eye differentiation. They may be involved in the epigenetic regulation of gene expression during development, as shown for the TIP60, DOM-A, and DOM-B [13,18], and their depletion may, in turn, cause up or down regulation of group of target genes, thus perturbing the genetic program required to achieve proper eye differentiation. This is also in accord with the finding showing that the INO80 remodeler can exert both positive and negative of control of homeotic gene expression in *D. melanogaster* [38].

Silencing of genes coding for YETI, BAP55, and YL-1 caused only minor eye defects with lower penetrance. Notably, silencing EAF6, GAS41, and MRG15 coding genes produced no obvious eye defects, but affected both viability and chromosome organization (Figure 2 and Figure 3, Table 1 and Table 3). It is then possible that EAF6, GAS41, and MRG15 may be not relevant, or even not essential, for eye development. Alternatively, it is possible that the composition of *D. melanogaster* chromatin remodeling complexes considered here (dTip60 or DOM-A.C and DOM-B.C) may differ between *in vivo* eye cells and cultured cell lines [13]. The last hypothesis is in accord with findings showing that during cellular differentiation, changes in subunit composition of chromatin remodeling complexes indeed play a critical role in establishing cell-type-specific transcriptional programs [39]. For example, BAF45a and BAF53a are found only in neural progenitors and exchanged for BAF45b and BAF53b in postmitotic neurons of human cells [40].

Several studies suggested an involvement of chromatin remodeling complexes in heterochromatin regulation. In mammals, the SWI/SNF-like protein SMARCAD1 promote the establishment of pericentric heterochromatin [41]. In yeast, the chromatin-remodeling factor FACT contribute to the organization of centromeric heterochromatin [42]. Moreover, mutations in genes coding for DOMINO, REPTIN, E(PC), MRG15 and GAS 41 subunits were found to be dominant suppressor of PEV [27,28,29,30]. Our results showed that *Bap55^EY1596^, Eaf6^d0660^,* and *Pontin^5.1^* mutant alleles are dominant suppressor of *In(1)wm4* variegation (Figure 5, Table 5).

Intriguingly, the *Dom3* allele is a dominant suppressor of *In(1)wm4* variegation, but fails to suppress the *7m27* variegation (Figure S2). This suggests that some subunits may play different roles in gene silencing which could be independent on the complex formation. It is possible that the different behavior of *Dom3* allele on two different variegated phenotypes depends on a differential recruitment of DOM proteins to constitutive heterochromatin regions. It can be envisaged that the Y chromosome short arm telomeric region where the 7m27 insert is located may be devoid of DOM proteins or may not be under their control. These results confirm and extend the findings showing that the dTip60 complex subunits, beside gene activation, are involved in epigenetic silencing [13,18,27,28,29,30].

The roles played in the epigenetic silencing by dTip60 (or DOM-A.C and DOM-B.C) subunits may also have an impact on the dynamic changes occurring in constitutive heterochromatin during development, which are involved in modulating heterochromatic gene expression Marsano, et al. [43]. Chromatin remodelers may act directly by binding heterochromatic DNA domains or alternatively their action could be mediated by other epigenetic factors. In accord, HP1a was found to interact with the YETI subunit [4] and with other chromatin remodeling factors [42].

## 4. Materials and Methods

### 4.1. Drosophila Strains and Genetic Crosses

Fly cultures and crosses were carried out at 25 °C in standard cornmeal yeast medium. The following stocks were obtained from the Bloomington Drosophila Stock Center: GAL4-driver lines, *In(1)wm4* (#807), *Dom3* (#9260), *Bap55^EY15967^* (#21174), *Eaf6^d0660^* (#19244), *Pontin^5.1^* (#64756) and YL-1 RNAi (#31938). MRG15-3xHA (#F003043), PONTIN-3xHA (F000819), DMAP1-3xHA (#F000742), and TIP60-3xHA (#F000567 and #F004945) were acquired from FlyORF center. The following RNAi-lines were acquired from the Vienna Drosophila Resource Center VDRC): Domino (#7787), Pontin (#105408), Reptin (#19021), Tip60 (#110617), E(Pc) (#35268), Bap55 (#24704) Yeti (#102960), Eaf6 (#101457), DMAP1 (#103734), MRG15 (#110618), and GAS41 (#12616). The *Su(var)205* and *7M27* lines were gifts by Sarah Elgin.

### 4.2. Cytology and Immunostaining

Polytene chromosome squashes and immunolocalization experiments were performed as described previously [4]. Salivary glands from 4–6 third instar larvae were used to prepare five slides and about 10 chromosome figures for each slide were examined, Two replicates were performed for a total of at least 100 figures. All slides were analyzed for chromosome morphology by two people and the results were averaged. Rat anti-Domino-A and B antibodies [25] were used at 1:500 dilution. Mouse anti-HA antibodies (Cell Signaling, #2367) were used at 1:100 dilution. Guinea pig anti-Tip60 antibodies [4] were used at 1:100 dilution. As secondary antibody, rat, mouse and guinea pig monoclonal Alexa-Fluor-conjugated antibodies (Life Technologies, Carlsbad, CA, USA) were used at 1:200 dilution. DAPI was used for DNA staining.

### 4.3. In Vivo Expression of HA-Fused Subunits or shRNAs

In yeast, the GAL4 transcription factor binds to the UAS regulatory sequences and activates expression of the gene downstream of UAS, which otherwise would be silent [24]. Using this system in *Drosophila melanogaster*, in vivo expression of transgenes can be performed in a spatiotemporal manner with suitable transgenic lines [43]. This approach allowed us to express HA-fused subunits or shRNA (short harpin RNA) to silence genes coding for the subunits under investigation ubiquitously during development (Table 1) or in specific tissues and organs (Figure 1, Figure 2 and Figure 3).

### 4.4. Western Blotting

*D. melanogaster* protein extracts were prepared in sample buffer from salivary glands. All the samples were loaded in a poly-acrylamide gel, transferred onto Polyvinylidene fluoride (PVDF) membrane and probed with mouse anti-HA (1:1000, Cell Signaling, Danvers, MA, USA, #2367) and mouse anti-α-Tubulin (1:5000, Sigma, St. Louis, MO, USA #T9026). The bands were immunodetected using the ECL kit from Thermo Scientific (Waltham, MA, USA).

### 4.5. RNA Extraction and Semi-Quantitative PCR

Total RNA was extracted from wandering third-instar larvae with the Trizol (EuroGold Trifast, EMR527100 Vetroscientifica, Rome, Italy) according to the manufacturer’s instruction. RNA was retrotranscribed with the RETROscript kit (AM1710; Fisher Scientfic, Pittsburgh, PA, USA), according to the manufacturer’s instruction. The reverse transcription was performed on a GeneAmp PCR System 2700 Thermal Cycler (Applied Biosystems, Foster City, CA, USA), according to the following PCR conditions: 25 °C for 10 s, 42 °C for 15 s, 85 °C for 5 s, 4 °C to ∞. Semi-quantitative PCR (sqPCR) reactions were conducted by using SapphireAmp Fast PCR Master Mix (Takara-bio, RR350B Saint-Germain-en-Laye, France) according to the manufacturer’s instruction. Gene-specific primers for sqPCR amplification were designed using Primer designing tool—NCBI—NIH (Table S1). Thermal cycling conditions were: 94 °C for 1 min; 30 cycles of 98 °C for 5 s, 55 °C for 10 s, and 72 °C for 10 s. The sqPCR amplification products were analyzed by electrophoresis in 1.5% agarose gel. Gene expression level was normalized to RPL32 ribosomal protein and quantified by ImageJ software. Values were calculated from three independent experiments.

### 4.6. Eye Pigment Assays

The extraction of red eye pigment was performed according to Ephrussi and Herold [44]. For each genotype, three replicate samples of 10 heads were performed Absorbance at 480 nm was then measured using a 96-well plate in a VICTOR Multilabel Plate Reader spectrophotometer (PerkinElmer, Waltham, MA, USA). Photographs of representative adult fly eyes were taken using a using a Nikon SMZ745T stereoscopic microscope (Minato, Tokyo, Japan) equipped with a digital C-mount camera. For both the eye pigment assay and the adult eye photographs, the appropriate balancer chromosome was used as the control.

### 4.7. Image Acquisition

DAPI stained and immunostained preparations were analyzed using a computer-controlled Nikon Eclipse 50i epifluorescence microscope equipped with UV-1A EX 365/10 DM 400 BA 400, FITC EX 465-495 DM 505 BA 515-555 and TRITC EX 540/25 DM 565 BA 605/55 filters using a plan achromat microscope objective 40X/0.65 WD 5.56 or 100XA/1.25 Oil OFN22 WD 0.2 objectives and QImaging QICAM Fast 1394 Digital Camera, 12-bit, Mono (Minato, Tokyo, Giappone). Images were imported into ImageJ software (http://rsbweb.nih.gov/ij/, accessed on 5 February 2021) and adjusted for brightness and contrast uniformly across entire fields where appropriate. Western blot digital images were acquired by Bio-Rad ChemiDoc MP Imager and analyzed by ImageLab software (Bio-Rad, Berkeley, CA, USA). Eye images were acquired using a Nikon SMZ745T stereomicroscope equipped with a digital C-mount camera. All the figures were constructed in Adobe Photoshop (San Jose, CA, USA).

### 4.8. Statistical Analysis

Data analyses were performed using the GraphPad Prism software (GraphPad Software, Inc., La Jolla, CA, USA). All results were expressed as mean ± SD values from three independent replicate experiments. *p* value of less than 0.05 (* *p* < 0.05, compared with the control group) were considered to be statistically significant by using two-tailed Fisher’s exact test.

## Figures and Tables

**Table 1:** Silencing of genes coding for Tip60 complex subunits arrests fly development. About 100 flies were scored for each cross. ^§^ Simultaneous silencing.

Subunit	Viability
BAP55	Early lethal
DMAP1	Early lethal
DOM-A/DOM-B ^§^	Early lethal
EAF6	Early lethal
E(PC)	Early lethal
GAS41	Late larval lethal
MRG15	Late larval lethal
NIPPED-A	Early lethal
PONTIN	Early lethal
REPTIN	Early lethal
TIP60	Early lethal
YETI	Late lethal
YL-1	Late lethal

**Table 2:** sqPCR quantification of silencing of genes coding for the subunits under investigation. The results are expressed as % mean ± SD values from three independent replicate experiments. ^§^ Simultaneous silencing.

	% Relative Intensity
control	100
DMAP1	22.9 ± 0.5
DOM-A and B ^§^	41.3 ± 1.3
EAF6	51.4 ± 0.6
E(PC)	32.1 ± 1.5
GAS41	14.1 ± 0.3
MRG15	28.5 ± 0.7
PONTIN	32.0 ± 0.3
REPTIN	29.4 ± 0.5
TIP60	56.0 ± 0.5
YETI	25.4 ± 0.7
YL-1	48.1 ± 0.3

**Table 3:** Quantification of polytene chromosome defects after silencing of genes coding for the subunits under investigation. About 100 chromosome figures were scored for each subunit. The results are expressed as % mean ± SD values from three independent replicate experiments. * = *p* < 0.05, compared to the control. ^§^ Simultaneous silencing.

	% Abnormal Chromosomes
control	1.8 ± 1.7
DMAP1	90.2 ± 9.9 *
DOM-A/DOM-B ^§^	94.6 ± 6.3 *
EAF6	28.1 ± 1.0 *
E(PC)	96.3 ± 1.3 *
GAS41	47.0 ± 14.4 *
MRG15	39.8 ± 0.7 *
PONTIN	92.5 ± 7.5 *
REPTIN	94.9 ± 4.4 *
TIP60	79.7 ± 13.3 *
YETI	94.8 ± 3.5 *
YL-1	31.8 ± 3.3 *

**Table 4:** Quantification of eye defects after silencing of genes coding for the subunits under investigation. About 60 flies were scored for each subunit. The results are expressed as % mean. *p* < 0.05 (*), compared to the control. ^§^ Simultaneous silencing.

	% Eye Defects
control	0
BAP55	8 *
DMAP1	42.42 *
DOM/DOM-B ^§^	100 *
EAF6	0
E(PC)	100 *
GAS41	0
MRG15	0
PONTIN	100 *
REPTIN	67.35 *
TIP60	67.52 *
YETI	1.51
YL-1	0

**Table 5:** Suppression of *In(1)w^m4^* variegation. The results are expressed as % mean ± SD values from three independent replicate experiments. * = *p* < 0.05, compared to the control.

Mutations	Control	Experimental
*Su(var)205*	0.038 ± 0.005	0.402 ± 0.008 *
*Domino^3^*	0.051 ± 0.028	0.249 ± 0.110 *
*Bap55^EY1596^*	0.033 ± 0.025	0.150 ± 0.039 *
*Eaf6^d06605^*	0.041 ±0.021	0.162 ± 0.061 *
*Pontin^5.1^*	0.043 ± 0.015	0.192 ± 0.066 *

## Data Availability

The data that support the findings of this study are available from the corresponding author upon reasonable request.

## Supplementary Materials

The following are available online at https://www.mdpi.com/article/10.3390/ijms22094525/s1, **Figure S1.** Eye color of *Bap55^EY1596^ Eaf6^d06605^*. Light-yellow eye color of A) *Bap55^EY1596^* and B) *Eaf6^d06605^* heterozygous mutations, **Figure S2.** The 7m27 variegated phenotype. (**A**) Variegated phenotype of 7m27; (**B**) one copy of *Su(var)205* suppresses 7m27; (**C**) One copy of *Dom3* allele does not suppress the variegated phenotype of 7m27, Table S1: Primer sequences used for reverse transcription-polymerase chain reaction (PCR).
