# Supplementary material for: In Vivo Silencing of Genes Coding for dTip60 Chromatin Remodeling Complex Subunits Affects Polytene Chromosome Organization and Proper Development in Drosophila melanogaster
Source: Int J Mol Sci. 2021 Apr 26;22(9):4525. doi: 10.3390/ijms22094525 (PMC8123692; doi:10.3390/ijms22094525)
Supplement: Supplementary file 1 [file ijms-22-04525-s001.zip › ijms-1191488-supplementary.pdf]

Supplementary materials

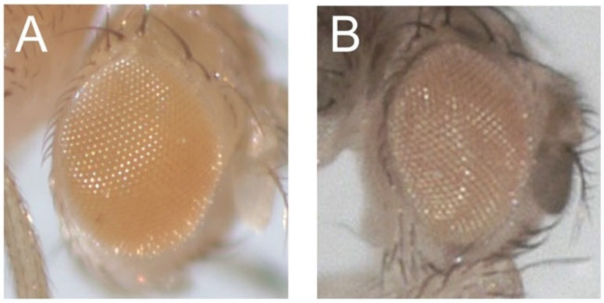

Figure S1. Eye color of *Bap55*^{EY1596} *Eaf6*^{d06605}**.** Light-yellow eye color of A) *Bap55*^{EY1596} and B) *Eaf6*^{d06605} heterozygous mutations.

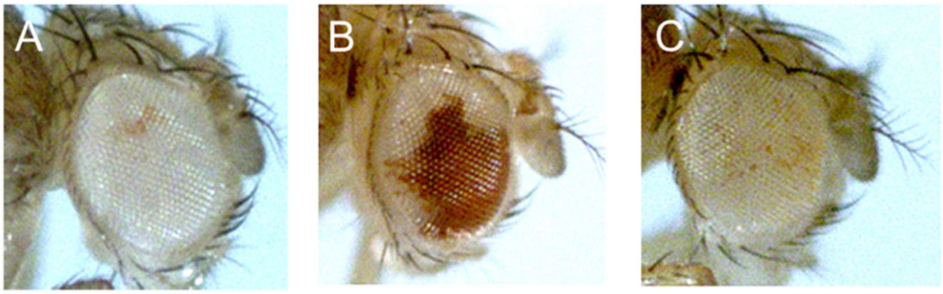

Figure S2. The 7m27 variegated phenotype**.** (**A**) Variegated phenotype of 7m27; (**B**) one copy of *Su(var)205* suppresses 7m27; (**C**) One copy of *Dom3* allele does not suppress the variegated phenotype of 7m27.

Table S1. Primer Sequences Used for Reverse Transcription-Polymerase Chain Reaction (PCR)

**Gene**	**Forward primer**	**Reverse primer**
domino	GACGGCGCTAGCTAAGGAAT	CCGCATCTTCGCTTTCTTCG
pontin	AAACCGAGGAGTATGTGCCG	CCATGTCGGCAGTGGAGTAG
reptin	GGAGATGAGCAAGACCGAGG	ATCTCTCCAGTATCGCCGGA
Tip60	GCGGCTTCCACATAGTAGGG	CGGCAATGAAGCACAATCTCT
E(Pc)	CCGTTACACACTGACCTCCC	TGGCGAAGATCAGCACAACT
Yeti	TATACGGAGGGAAACGCAGC	TCGACGTTGGTTAAGCCGTT
YL-1	GTGGAGGACTACATGCCCAC	TATGGCTGCTGTGTGATCGG
Eaf6	GTTGGCGGACCTCATCAAGA	ACACCATTCCGCTCACTACG
DMAP1	GATACCGCTCTGGGAATCGG	TCTCCTCCTCCACCTGTTGT
Mrg15	ACCGCCAACTCTACAACCAG	TGCGCCAAATACTGCTCAGA
Gas41	ACGAGATTACCGAGACCGGA	ACCTTCGCCAATTCTGCCTT
Rpl32	GCCCAAGGGTATCGACAACA	CTTGCGCTTCTTGGAGGAGA
